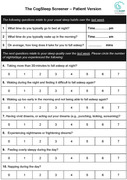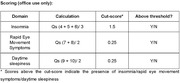# Validating the CogSleep Screener in older adults at a memory and cognition clinic

**DOI:** 10.1002/alz.086428

**Published:** 2025-01-09

**Authors:** Shawn Dexiao Kong, Zoe M Schrire, Pinghsiu Lin, Simone Simonetti, Nathan Cross, Loren Mowszowski, Sharon L Naismith, Healthy Brain Ageing Research Clinic

**Affiliations:** ^1^ The University of Sydney, Camperdown, NSW Australia; ^2^ Healthy Brain Ageing Program, Brain and Mind Centre, University of Sydney, Camperdown, NSW Australia; ^3^ School of Psychology, Faculty of Science, University of Sydney, Sydney, NSW Australia; ^4^ Charles Perkins Centre, University of Sydney, Sydney, NSW Australia; ^5^ Healthy Brain Ageing Program, Brain and Mind Centre, University of Sydney, Sydney, NSW Australia; ^6^ University of New South Wales, Sydney, NSW Australia; ^7^ Brain and Mind Centre, University of Sydney, Sydney Australia; ^8^ School of Psychology, Faculty of Science, University of Sydney, Sydney Australia; ^9^ Healthy Brain Ageing Program, Brain and Mind Centre, University of Sydney, Sydney Australia; ^10^ Healthy Brain Ageing Program, University of Sydney, Sydney Australia; ^11^ University of Sydney, Sydney, NSW Australia; ^12^ The University of Sydney, Sydney, NSW Australia

## Abstract

**Background:**

While sleep disturbances are prevalent in older people and are linked with poor health and cognitive outcomes, screening for the range of sleep disturbances is inefficient and therefore not ideal nor routine in memory and cognition clinic settings. We aimed to develop and validate a new brief self‐report questionnaire for easy use within memory and cognition clinics.

**Method:**

Older adults (N = 402, mean age 67.3 years, range 50‐86, 63.6% female) were recruited from a memory and cognition research clinic. All participants completed a comprehensive medical, neuropsychological and mental health assessment, alongside self‐report instruments, including existing sleep questionnaires and a new 10‐item sleep questionnaire, the CogSleep Screener. We examined the factor structure, convergent validity, internal consistency, and discriminant validity of this novel questionnaire.

**Result:**

Using exploratory principal component analysis, a 3‐factor solution was generated highlighting the factors of *Insomnia, Rapid Eye Movement (REM) Symptoms* and *Daytime Sleepiness*. Each factor was significantly correlated with currently used sleep questionnaires for each subdomain (all Spearman rho>0.3, all p<.001), suggesting good convergent validity. Internal consistency was also good (Crobach’s α = .73). Receiver operating characteristic curves showed good discriminative ability between participants with and without sleep disturbances (all area under curve>0.7, all p<.01).

**Conclusion:**

The CogSleep Screener has good psychometric properties in older to elderly adults attending a memory and cognition clinic. The instrument has the potential to be used in memory clinics and other clinical settings to provide quick and accurate screening of sleep disturbances.